# A global community of practice to improve public–private engagement in TB: the TBPPM Learning Network

**DOI:** 10.5588/ijtldopen.26.0246

**Published:** 2026-10-12

**Authors:** P. Heitkamp, S. Sheokand, J.S. Klinton, V. Yellappa, M. Putturaj, R. Ananthakrishnan, A. Akinrin, P. Thapa, M. Pai

**Affiliations:** 1 Research Institute of the McGill University Health Center, McGill University, Montreal, QC, Canada;; 2 The TBPPM Learning Network, McGill University Health Center, Montreal, QC, Canada;; 3 KNCV TB Plus, The Hague, Netherlands;; 4 India Chapter, The TBPPM Learning Network, McGill University Health Center, Montreal, QC, Canada;; 5 IPH Bengaluru, Bengaluru, India;; 6 REACH, Chennai, India;; 7 Nigeria Chapter, The TBPPM Learning Network, McGill University Health Center, Montreal, QC, Canada;; 8 School of Population and Global Health, McGill University, Montreal, QC, Canada;; 9 Department of Medicine Solna, Karolinska Institutet, Stockholm, Sweden.

**Keywords:** tuberculosis, health systems, private sector, knowledge exchange, capacity building, implementation

## Abstract

**BACKGROUND:**

In many high-TB-burden settings, most people seek initial care from private providers who are often not integrated into national TB programmes. Public–private mix (PPM) strategies are therefore critical, yet difficult to operationalise. Communities of Practice (CoPs) can act as health system enablers and facilitate knowledge exchange, but empirical evidence on their long-term function in disease specific contexts is limited.

**METHODS:**

We conducted a mixed-methods descriptive analysis of the TBPPM Learning Network (LN) from 2019 to 2025. Quantitative data from routine platform analytics were triangulated with thematic analysis of 53 webinars and 9 Friday Forum topics.

**RESULTS:**

The LN engaged over 6,000 TB professionals across more than 130 countries, with over 75% from low- and middle-income countries. It adapted rapidly during the COVID-19 pandemic, strengthened South–South exchange, and supported leadership development among frontline implementers. Six key lessons were identified, including the importance of sustained facilitation, community-building, and adaptability, alongside persistent challenges in translating learning into action in resource-limited settings.

**CONCLUSION:**

A global virtual CoP can function as a scalable mechanism to support knowledge translation, capacity strengthening, and collaboration in complex TB health systems, but sustained investment is required to translate learning into policy and practice.

TB remains a major global health challenge despite recent progress.^1^ In seven high-TB-burden countries with a large private sector, 67%–85% of patients first seek care from private or informal providers.^2^ However, these providers are often not integrated into national TB programmes as they account for only 14%–32% of the TB notifications leading to gaps in notification, delays in diagnosis, and inconsistent quality of care.^2^ Public–private mix (PPM) is therefore critical to improving TB outcomes. Structured approaches to engage private providers have demonstrated improvements in case detection, treatment initiation, and follow-up.^3,4^ Communities of Practice (CoPs) are recognised as platforms for knowledge exchange, peer learning, and collaboration across sectors. While CoPs have been applied in various global health domains,^5–7^ there is limited empirical evidence on how virtual, disease-focused CoPs function over time in mixed public–private health systems. The TBPPM Learning Network^8^ (LN) was established in 2019 as a global virtual CoP to support knowledge exchange, build capacity, and strengthen collaboration among stakeholders engaged in TB care. The LN was set up to provide peer-to-peer learning, identify champions, especially from high-TB-burden settings, and share lessons and best practices on engaging all providers (including private and informal) in TB care. Activities include webinars, case studies, forum discussions, a library, and an online platform with details described in later sections. The LN intentionally helped create and even provided partial funding for country learning networks in India and Nigeria to facilitate localised engagement and ownership.

This study examines the evolution, outputs, and lessons from a global virtual CoP over 6 years, with the aim of informing the design and application of CoPs in TB and other health systems.

## METHODS

We conducted a mixed-methods descriptive analysis of the LN between 2019 and 2025, using routinely collected platform data and qualitative thematic analysis. The analysis was guided by Burr’s framework for a virtual CoP.^9^ It categorises user interaction into three dimensions: 1) system interaction (platform engagement); 2) content interaction (learning materials/knowledge product engagement); and 3) learner interaction (peer-to-peer engagement).^9,10^ This framework was used to categorise and interpret patterns of engagement and outputs across the LN.

Quantitative data included membership statistics, webinar registrations and attendance, course participation, and engagement metrics from the online platform. Table 1 outlines the monitoring framework used to routinely collect data and indicators. These data were used to assess reach, growth, and patterns of participation. Qualitative data were derived from a thematic review of 53 webinars and 9 discussion forums conducted between 2019 and 2025. Topics were coded inductively by the author team to identify patterns in content evolution, engagement priorities, and emerging themes. The analysis aimed to describe patterns of engagement, outputs, and thematic evolution over time.

**Table 1.** Monitoring and evaluation indicators used for sourcing quantitative analytics data between 2019 and 2025.

Monitoring framework: TBPPM Learning Network			
Measures	Indicators	How to measure	Frequency of tracking
Community analytics	How many members are part of the TBPPM Learning Network?	Hivebrite^A^ analytics	Fortnightly
	How many members have uploaded their profile picture?		
	How many members are from the ‘Big Seven’?		
	How many members are from the ‘Other PPM Priority Countries’?		
	How many members represent each industry? (How do you identify yourself within the TB community?)		
Content analytics	How many articles were shared by admin and users?	Hivebrite analytics + manual	Monthly
	How many events were created by admin and users?		
	How many job postings were created by admin and users?		
	How many useful interactions happened on the website?		
Website analytics	How many people visited tbppm.org?	Google analytics	Monthly
	What is the average time users spent on the website?		
	What was the most visited page in the month?		
	Which page did users commonly enter the website through?		
Social media analytics	How many people follow our social media account?	Social media data/manual	Monthly
	How many people in our mailchimp database?	Mailchimp/manual	
Publication analytics	How many peer-reviewed publications/opinions pieces facilitated?	Output	Product based
	How many peer-reviewed publications/opinions pieces led?		
Feature stories analytics	Country representation and topics covered	Output	Product based
Webinar analytics	Gender, country representation, implementers, and topics covered	Output	Event based
Country chapters analytics	Number of chapters: activities, output/outcomes	Output	Quarterly

A TBPPM Learning Network website hosting platform.

### Ethical statement

As the study relied on aggregated programmatic data without individual-level identifiers, ethical approval was not required.

## RESULTS

Our synthesised interpretation of Burr’s three dimensions is placed in the centre of our analysis (Figure 1), visualising the three dimensions of interaction (system, content, and learner) representing the movement of each user in a fluid, non-sequential way. This representation recognises the engagement of community members at different levels and at different times – some interact mainly with the platform itself (system interactions), others engage with learning materials and moderators (content interactions), while others might engage in peer-to-peer exchanges that build community (learner interactions). The middle circle represents the key outputs of the LN and the outer circle notes the ultimate impact in the real world.

**Figure 1.**
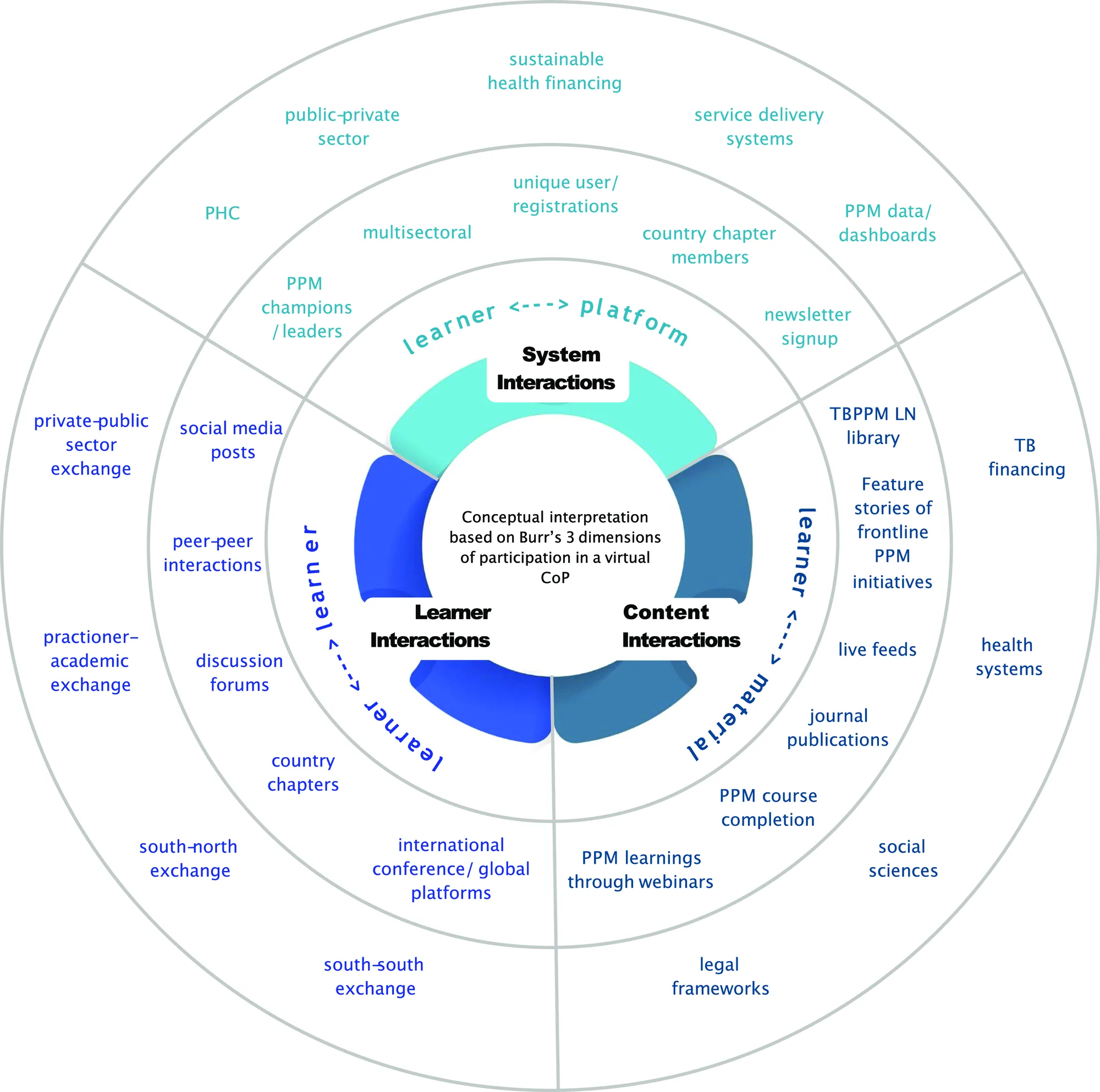
Dimensions of user interaction within the TBPPM LN. PHC = primary health care; PPM = public–private mix; LN = Learning Network.

### System interaction: reach, growth, and engagement

The LN expanded from about 100 members in 2019 to over 3,100 registered members by 2025, with additional reach of over 6,000 individuals through newsletters and events. Most people were invited to join the LN through social media and following participation in webinars. Figure 2 illustrates steady growth in membership and geographic reach over time. Members represented more than 130 countries with over 75% from low- and middle-income countries, mostly representing TB programme managers in non-governmental organisations and government bodies, project staff, and researchers. Participation diversified over time, with increased representation of frontline implementers, including providers, civil society organisations, and community stakeholders. From 2023, the LN identified a cohort of 193 champions which has accumulated by the end of 2025 into a total of 243 ‘PPM Champions’ and 165 ‘PPM Leaders’. Two country chapters (India and Nigeria) were established to support localised engagement in support of national and state TB programmes, with membership growth, structured PPM coordination at national level, and input on programmes and policy.

**Figure 2.**
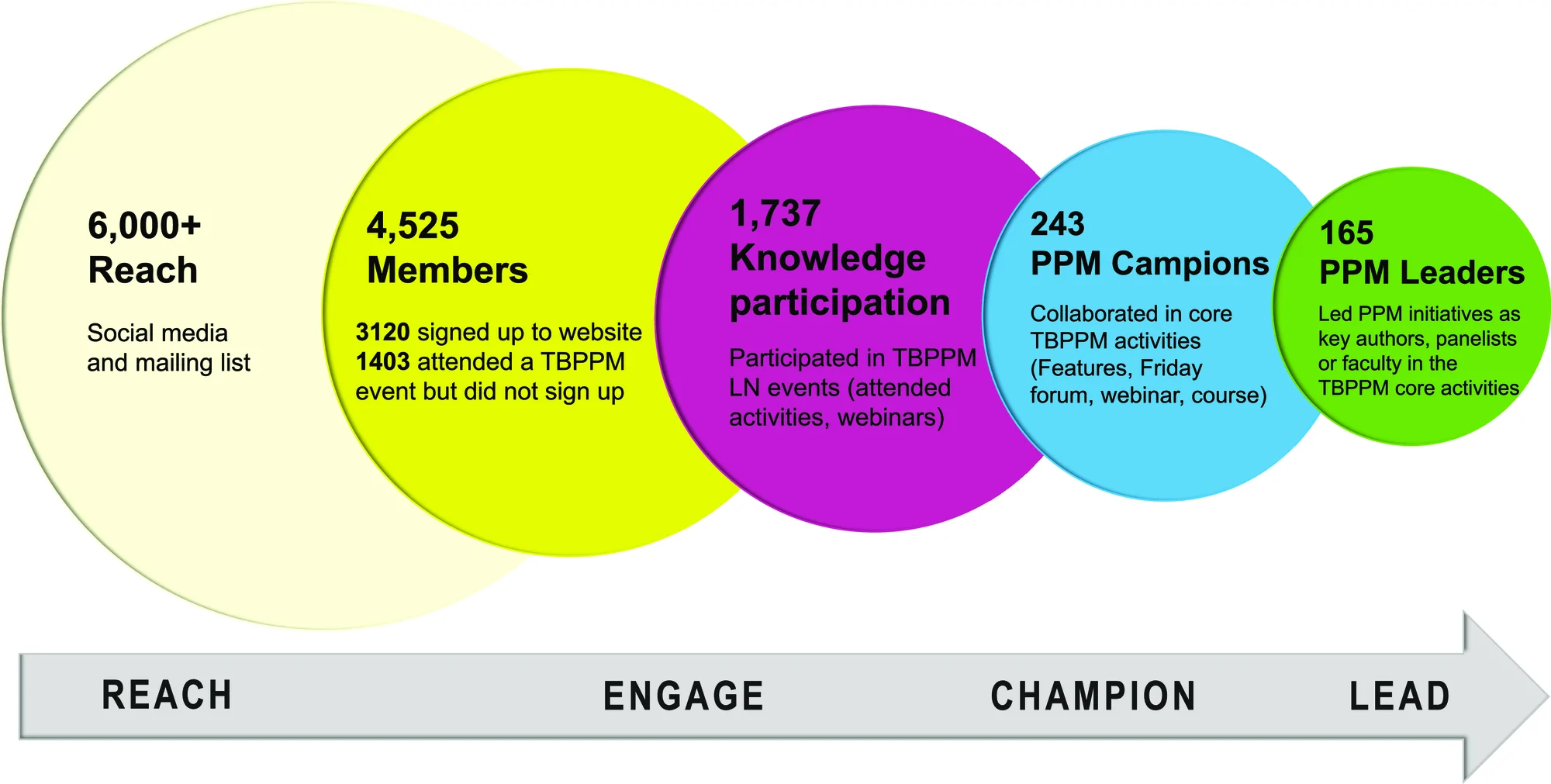
Reach of TBPPM LN (2019–2025). PPM = public–private mix; LN = Learning Network.

### Content interaction – knowledge sharing and learning outputs

Between 2019 and 2025, the LN delivered 53 webinars and 9 Friday Forums attracting approximately 11,000 registrations and over 22,000 views. The platform hosts more than 1,450 resources, including guidelines, feature stories and case studies, and implementation tools. The LN functions as a broader knowledge-sharing space, and a structured training course on public–private engagement was introduced in 2022 on the LN platform and repeated over the years through the McGill Summer Institutes in Global Health, reaching 350 participants from 38 countries.^11^ Course completion exceeded 70%, and participants included policymakers, providers, and researchers. The LN also contributed to knowledge generation and dissemination through feature stories^12^ (41 unique frontline projects), peer-reviewed publications^13–23^ (16 articles), and collaboration with global stakeholders.^2,24–26^

### Learner interaction – collaboration and community-building

The LN facilitated peer-to-peer exchange through online forums (98 topics) generating 200+ comments from members. The website’s ‘live feed’, which functions as a social media interaction page, has 1,500+ posts that are disseminated on a weekly basis in emails to all members. Country-level activities demonstrate how network connections translate into local initiatives and eventually policy influence. Examples are the ‘Compendium of Public Private Mix for Tuberculosis Control in India: Accelerating Efforts to End Tuberculosis’ highlighting effective PPM approaches, and three short films offering a unique virtual field visit to successful PPM models in action.^27^ In 2023, the India country chapter set up a national secretariat^28^ hosted by the Institute of Public Health (IPH) in Bengaluru, India, and with a multisectoral advisory board, chaired by REACH (an organisation with over 20 years of TB and PPM knowledge), reinforcing local leadership and sustainability. In Nigeria, the network contributed to policy dialogue and strategy development for private sector engagement, through close collaboration with the national PPM steering committee. The two country chapters underwent an independent evaluation providing insights from local communities on the progress made over 5 years.^29^

### Thematic evolution

Thematic analysis of webinars and forums showed progression in focus areas over time. Figure 3 represents the evolution of thematic priorities across LN’s development phases. In 2020–2021, discussions centred on maintaining TB services during the COVID-19 disruptions.^23^ In 2021–2022, the focus shifted to formalising public–private engagement, including laboratory and pharmacy integration.^30,31^ By 2023, discussions emphasised localisation of knowledge, country ownership, and operational challenges, including treatment regimens and patient-centred care.^17,22^ In 2024–2025, themes evolved towards health systems integration, financing, and sustainability.^32,33^

**Figure 3.**
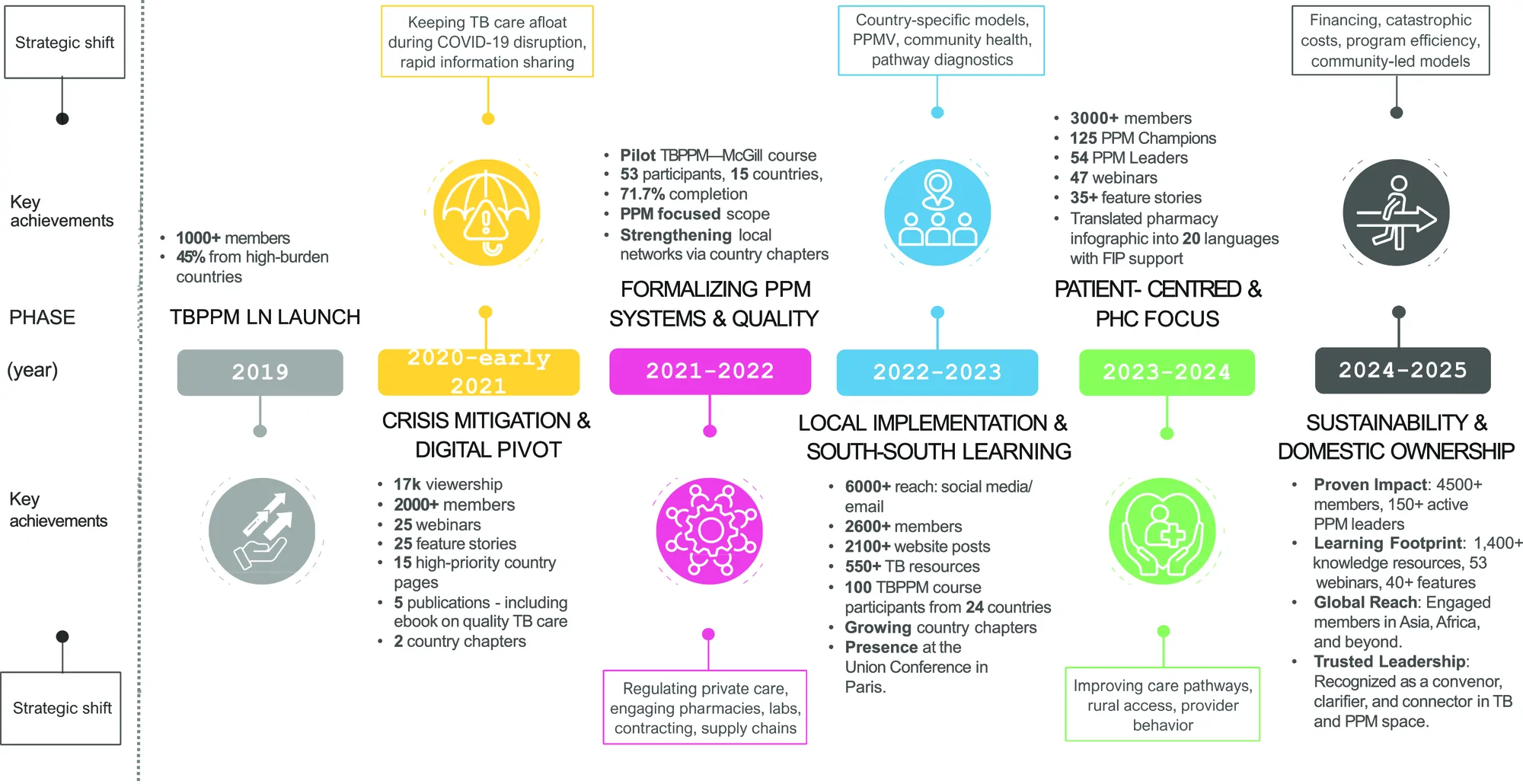
TBPPM LN – growth phases and strategic shifts in knowledge products (2019–2025). PPM = public–private mix; LN = Learning Network.

### Lessons learned

The following six lessons emerge from the LN’s 6-year experience (Table 2), which highlight both the strengths and inherent challenges of sustaining a global virtual CoP. First, amplifying the voices of frontline implementers and community actors in high-TB-burden countries strengthened inclusivity and contributed to more equitable, multisectoral engagement; however, participation required sustained effort, as many implementers often from civil society had limited time and resources to engage consistently. While strengthening health systems is unique to each country, for example, in some countries public capacity on private contracting takes priority, in others streamlining communication with private sector, the LN supports civil society and frontline implementers. Second, effective facilitation proved essential in building trust and long-term linkages, particularly during periods of disruption such as the COVID-19 pandemic, yet this role remained resource-intensive, largely invisible, and difficult to sustain without dedicated investment. Third, community-building emerged as a critical foundation for sustainability, with relational outcomes such as trust, peer credibility, and shared identity (‘soft architecture’) driving long-term engagement; however, these outcomes were difficult to measure, standardise, or translate into formal guidance. Fourth, while the LN successfully generated and disseminated knowledge, translating this learning into policy and practice remained a persistent challenge, particularly in the absence of structured agendas, country-level resources, and accountability mechanisms, highlighting the risk of remaining a ‘knowledge-sharing’ platform without sufficient action pathways. Fifth, efforts to dissolve traditional hierarchies and promote equal partnerships fostered dialogue and collaboration across sectors, but entrenched power dynamics and structural incentives continued to limit full integration of private and community actors. Finally, the LN’s adaptability, especially its rapid shift to digital engagement, enabled scale, inclusivity, and continuity, yet digital exclusion and the limited depth of virtual interaction underscored the need for hybrid models and localised engagement.

**Table 2.** Lessons learned based on the outcomes from the TBPPM LN (2019–2025).

	Lesson	What worked	What did not work
1.	Amplify implementer and community voices	Survey feedback shows that elevating the voices of frontline implementers and grassroots actors in private provider engagement worked.	Majority of implementers able to engage voluntarily were CSOs and non-profit organisations (often lacking time for sustained engagement according to annual surveys).
		The LN has contributed to an enabling environment for equitable partnerships and inclusive multisectoral decision making.	Sharing and documenting lessons remained a luxury, particularly in the absence of resources for operational research, technical assistance, or knowledge translation support.
		PPM insights have informed TB programmes while advancing PHC integration – especially relevant as new point-of-care diagnostics emerge.**^A^**	
Key Learning: Without financial or strategic incentives, maintaining continued engagement is challenging.			
2.	Stay persistent and flexible as facilitators	Effective convening, connecting, and facilitation was supported by an ongoing presence and a neutral stance across diverse actors.	Facilitation is time-intensive and often invisible. Success meant partners stories and country outputs (not the Team behind the LN) were in the foreground, making contribution and credit difficult to attribute.
		Persistence helped build trust and strengthen long-term linkages within PPM ecosystems, particularly during difficult transitions such as the COVID-19 pandemic.	
Key Learning: Facilitation requires long-term investment, yet human resource and the secretariat function is the least funded and the hardest to justify without clear metrics of impact.			
3.	Build communities and adapt to local contexts for sustainability	Community-building was essential for sustaining relationships, catalysing dialogue, and improving responsiveness to context-specific challenges.	Expansion of membership in national chapters requires strategy and time: deliberate mapping of membership gaps, active outreach/follow-up to new organisations, etc.
		Most meaningful gains were relational: trust, peer credibility, shared identity, and sustained engagement – what governance literature refers to as the ‘soft architecture’ of systems.**^B^**	High staff turnover in member organisations, limited digital access, and fragmented private sectors made sustainable engagement in the global LN beyond individual webinar participation hard.
		These outcomes supported long-term sustainability even without formal structures.	
Key Learning: Relational outcomes are difficult to standardise, measure, or compare across settings. They do not fit traditional monitoring frameworks but are context-specific.**^C^**			
4.	Translate knowledge into action	The LN demonstrated the importance of ‘change activism’ – moving from knowledge sharing to evidence-based action.	The LN did not formalise a global strategic policy agenda with milestones, PPM-specific targets, or a structured action pathway following learnings.
		The LN Knowledge system (pulling in knowledge, processing, providing, pushing out, prioritising learning) became a functional framework.	Without country-level resources for policy and implementation, many discussions remained conceptual. As seen in other public–private dialogues, there was a risk of becoming a ‘talk shop’ without structured follow-through.**^D^**
		Inclusion of grassroots implementers ensured data and insights remained grounded in reality, including real-time stories and practical evidence to guide adaptive responses.	
Key Learning: To move from knowledge into action or policy change, dedicated stakeholders and resources need to be included at global and country level.			
5.	Dissolve hierarchy and promote equal partnerships	The LN helped challenge traditional hierarchies in TB – long criticised as highly vertical on the neutral platform.	Dismantling entrenched hierarchies proved difficult in many country contexts.
		Surveys indicate change of attitudes to private sector engagement in TB. Peer connections and cross-learning helped foster public–private dialogues, more collaborative systems, and built momentum for multisectoral partnerships needed to End TB.	The LN funded by Gates and hosted by McGill (both North-based institutions) are an illustration that power asymmetries persist in global health.
Key Learning: Without systemic changes to include new stakeholders (private sector, South–South, multiple sectors at PHC) limited progress will be seen. Similar challenges in intersectoral partnerships are widely documented.**^E^**			
6.	Stay adaptable and seize opportunities in digital innovation	The LN’s resilience was evident during the pandemic.	Digital exclusion limited equitable participation for implementers without reliable connectivity, particularly in rural or underserved areas.
		Rapid digital transition sustained and expanded engagement, enabling participation from frontline providers, students, smaller NGOs, and community groups.	Virtual platforms could not fully replace the depth of in-person field learning and relationship-building.
		Recorded content created a permanent, open-access knowledge library. Hybrid and asynchronous options later preserved participation as countries returned to offline work.	As participation grew, expectations also expanded, risking mission drift without additional resources.
		The LN responded in real time to emerging needs – highlighting digital tools, private pharmacy engagement, supply chain disruptions – and catalysed a TBPPM course and formal country chapters.	
Key Learning: Sustaining engagement in a virtual CoP is challenging without in-person interaction, underscoring the need for localised communities of practice and hybrid formats, alongside better support for implementers with limited digital access.			

CSO = civil society organisation; PHC = primary health care; PPM = public–private mix; LN = Learning Network; CoP = Community of Practice; NGOs = non-governmental organisations.

A Reference ^32^.

B World Health Organization. Engaging the private health service delivery sector through governance in mixed health systems: strategy report of the WHO Advisory Group on the Governance of the Private Sector for Universal Health Coverage. Geneva: WHO, 2020.

C Reference ^29^.

D O’Hanlon B, et al. Public–private dialogue: an essential approach to engage non-state actors in a mixed health system. BMJ Glob Health. 2025;8(Suppl 5):e014699.

E Aivalli P, et al. Power dynamics and intersectoral collaboration for health in low- and middle-income countries: a realist review. Health Policy and Planning. 2025;40(6):661–683.

Together, these findings illustrate that while virtual CoPs can be powerful enablers of knowledge exchange and system connectivity, their impact depends on sustained investment, intentional design for action, and integration with local health system structures.

## DISCUSSION

This study provides empirical evidence on the role of a global virtual CoP in supporting public–private engagement in TB care. Over 6 years, the LN demonstrated the ability to sustain participation across diverse settings, adapt to external shocks such as the COVID-19 pandemic, and support knowledge exchange and leadership development.

The findings highlight that CoPs can function as health system enablers by connecting stakeholders, facilitating shared learning, and strengthening capacity. The LN’s evolution (from crisis response to system-level discussions) illustrates how CoPs can mature over time. Reflecting on the reasons why the LN was set up in 2019, it has solidified its role as a connecting force in the global TB response. Over 6 years, it has brought in new stakeholders, strengthened knowledge on PPM through courses and resources, diversified engagement in its 6,000+ wide network, and consistently amplified implementers and community voices in driving people-centred engagement in TB care. Compared to the general notion that larger CoPs have a 90-9-1 profile (90% periphery; 9% active; 1% core),^34^ which is comparable with the LN ‘reach-engage-lead’ paradigm, it is clear that the LN has been able to build a stronger community with more engaged and leading participation. By showcasing real-world public–private implementation models and recognising country level champions, the LN has helped bridge the gap between evidence, policy, and practice. Given the vastness of all the knowledge products available on the LN, materials can be used for further research and complemented by qualitative data in the future to provide insight into private sector engagement (PSE) to strengthen integrated services at primary health care levels.

However, several limitations were identified. First, the study relies on descriptive data and cannot establish causal relationships between network activities and health outcomes. Second, engagement levels varied, and translating knowledge into policy or practice remained a challenge. Third, digital platforms may exclude participants with limited access to technology. Fourth, sustainability is hard to achieve and not consistently defined. Despite these limitations, the study suggests that investing in CoPs can strengthen relational and organisational aspects of health systems, which are often overlooked in traditional programme approaches. These findings suggest that CoPs can function as ‘soft infrastructure’ supporting coordination and capacity in fragmented health systems. Similar models could be applied to other health areas requiring multisectoral coordination.

Overall, sustainability remains a key challenge for virtual CoPs, particularly in the context of declining global health financing. The LN was funded by the Gates Foundation, with sustainability as a specific aim of the second grant. Sustainability in general refers to the long-term maintenance of communities, social institutions, and societal practices. In project terms, this is mostly interpreted as continuation of funding. Given the financial strain in global health due to widespread international aid cuts, there was limited scope in financing new coalitions to continue the LN community and activities. Yet, sustainability can also be seen in the connections and PPM knowledge left behind, the changed attitudes or practices, and collaborative culture in the TB community. Many of the existing partners remain and continue to collaborate, united by private sector engagement as an important field to explore the next phase. Just as we are looking to transition from global aid to sustainable domestic financing, we also need to look for transitions of global initiatives to South–South-led networks driven by countries with high TB burden.^33^ Sustainability for CoPs could look like ‘hosting’ arrangements in South-based academic institutes as we now have in India. The India LN, hosted by IPH, is a great example of win-win in sustaining focus on private sector engagement in TB, embedded in health systems research while highlighting TB needs.

## CONCLUSION

The TBPPM Learning Network has provided lessons about the functioning of a virtual CoP that works beyond geographical borders facilitating continuous peer-to-peer knowledge exchange, capacity building, and collaboration in complex TB health systems, but requires sustained investment to translate learning into action. A virtual community can accelerate adoption and scale-up of best practices and also save time spent on testing what may work and not work in diverse domestic settings. The LN demonstrates how virtual CoP can function as durable health system enablers – connecting evidence to action, amplifying frontline voices, and contribute to accelerating equitable TB care in complex mixed health systems.
